# COMPARISON BETWEEN OSTOMY CLOSURE USING PURSE-STRING VERSUS LINEAR IN CHILDREN

**DOI:** 10.1590/0102-672020220002e1709

**Published:** 2022-12-19

**Authors:** Shahnam Askarpour, Mehran Peyvasteh, Farbod Farhadi, Hazhir Javaherizadeh

**Affiliations:** 1 Pediatric Surgery, Ahvaz Jundishapur University of Medical Sciences – Ahvaz, Khouzestan, Iran;; 2 Alimentary Tract Research Center, Clinical Sciences Research Institute, Ahvaz Jundishapur University of Medical Sciences – Ahvaz, Khouzestan, Iran.

**Keywords:** Colostomy, Postoperative Complications, Infections, Infant, Newborn, Child, Colostomia, Complicações Pós-Operatórias, Infecções, Recém-nascido, Criança

## Abstract

**BACKGROUND:**

Type of ostomy closure has connection with some complications and also cosmetic effects.

**AIMS:**

This study aimed to compare result of colostomy closure using purse-string method versus linear method in terms of surgical site infection, surgical time, and patient satisfaction.

**METHODS:**

In this study, 50 patients who underwent purse-string ostomy closure and 50 patients who underwent linear closure were included. Two groups were compared for surgical time, wound infection, patient satisfaction, scar length. A p-value <0.05 was considered significant.

**RESULTS:**

Wound infection was not reported among purse-string group compared to 10% in linear group (p=0.022). Scar length was 24.09±0.1 mm in purse string and 52.15±1.0 mm in linear group (p=0.033). Duration of hospital admission was significantly shorter in purse-string group (6.4±1.1 days) compared to linear (15.5±4.6 days, p=0.0001). The Patient and Observer Scar Assessment Scale scale for observer (p=0.038) and parents (p=0.045) was more favorable among purse-string group compared to linear.

**CONCLUSION:**

Purse-string technique has the less frequent surgical site infection, shorter duration of hospital admission, less scar length, and more favorable cosmetic outcome, compared to linear technique.

## RESUMO

**RACIONAL:**

A técnica de fechamento da ostomia tem relação com algumas complicações e também efeitos estéticos.

**OBJETIVOS:**

Comparar o resultado do fechamento da colostomia pelo método em bolsa *versus* método linear, em termos de infecção do sítio cirúrgico, tempo cirúrgico e satisfação do paciente.

**MÉTODOS:**

Foram incluídos 50 pacientes que não realizaram o fechamento da estomia em bolsa e 50 pacientes que foram submetidos ao fechamento linear. Os dois grupos foram comparados quanto ao tempo cirúrgico, infecção da ferida, satisfação do paciente, comprimento da cicatriz. Valor de p menor que 0,05 foi considerado significativo.

**RESULTADOS:**

A infecção da ferida não foi registrado no grupo de bolsa, em comparação com 10% no grupo linear (p=0,022). O comprimento da cicatriz foi de 24,09±0,1 mm no grupo de bolsa e 52,15±1,0 mm no grupo linear (p=0,033). O tempo de hospitalização foi significativamente menor no grupo em bolsa (6,4±1,1 dias) em comparação ao linear (15,5±4,6 dias, p=0,0001). A escala Patient and Observer Scar Assessment Scale para observador (p=0,038) e pais (p=0,045) foi mais favorável entre o grupo em bolsa, em relação ao linear.

**CONCLUSÕES:**

A técnica em bolsa apresentou infecção do sítio cirúrgico menos frequente, menor tempo de internação, menor comprimento da cicatriz e resultado cosmético mais favorável, em comparação com a técnica linear.

## INTRODUCTION

Anorectal malformations and Hirschsprung disease are the main indications for ostomy formation among neonates and children^3^. After ostomy formation, another challenge is ostomy closure. Closure type has connection with some complications and also cosmetic effects. In more than 41% of children with ostoma, wound infection was reported^18^. Following ostomy closure, complications such as obstruction, infection, and necrosis may occur^1,25^.

Many studies exist in relation to wound infection and length of hospitalization^13^. McCartan et al. reported wound infection significantly reduced in purse-string method^18^. Reid et al. reported that, among 30 purse-string methods, 2 (6.7%) showed wound infection and, in linear, 38.7% had it^21^. According to systematic reviews, purse-string resulted in lowering wound infection, but about hospitalization more research were suggested^16,18,22^. Sureshkumar et al., on antibiotic treatment, showed wound infection and duration of antibiotic treatment significantly lower in purse-string compared to linear^23^. In contrary, Lee et al.^14,15^ showed that purse-string technique was associated with lower rate of infection but with longer healing time compared to linear closure. Han et al. modified that purse-string ostomy closure was associated with lower rate of wound infection, less hospital stay, and lower cost of hospitalization compared to linear closure, but with longer wound healing^9^. In the systematic review by Gachabayov et al.^7^, purse-string technique was associated with less infection compared to linear for reverse ileostomy, and Juratli et al.^11^ refer lower incidence of incisional hernia.

Limited studies have shown cosmetic effect, patient satisfaction, and wound infection based on method recommendation^6,20^.

The objective of this study was to compare purse-string ostomy closure versus linear closure in terms of wound infection, duration of hospitalization, and cosmetic effect.

## METHODS

This is a clinical trial registered at IRCT numbered IRCT20121010011068N3 and approved by Ethical Committee of the University. Children were randomly assigned in case or control group. Informed consent was signed by parents or legal guardians.

Children referred to Hospital for colostomy closure were included. A total of 100 patients were included (linear group=50, purse-string=50). Inclusion criterion was age 0–6 years old. Exclusion criteria were age >6 years, dead cases, patient incompliance, speech problem, and brain problem. Patients were randomly placed in purse-string (case) and linear (control) groups.

Follow-up period was 1 year following surgery. Follow-up was done on 1, 2, and 3 days after surgery; discharge; 30 days; and 6 and 12 months after surgery. Follow-up method was by phone and follow-up visit. The Patient and Observer Scar Assessment Scale (POSAS) was used for scar evaluation^4,5^.

### Statistical analysis

Data analysis was done using the t-test and Mann-Whitney U test. A p-value <0.05 was considered significant.

## RESULTS

As scheduled, colostomy closure using purse-string was done in 50 cases and linear in another 50. Gender, type of colostomy, and indications for colostomy are mentioned in Table 1.

**Table 1:** Gender, type of colostomy, and indication for colostomy between two groups.

		Linear n (%)	Purse-string n (%)	p-value
Gender	Male	33 (66%)	32 (64%)	0.834
	Female	17 (34%)	18 (36%)	
Age (months)		13.4±6.5	14.9±6.5	0.277
Type of colostomy	Diverting colostomy	15 (30%)	16 (32%)	0.829
	Loop colostomy	35 (70%)	34 (68%)	
Colostomy indication	Anorectal malformation	28 (56%)	31 (62%)	0.872
	Hirschsprung disease	7 (14%)	7 (14%)	
	Imperforate anus	10 (20%)	9 (18%)	
	Colonic atresia	5 (10%)	3 (6%)	

Wound infection was not reported among purse-string group compared to 10% among linear (p=0.022) (Table 2).

**Table 2:** Comparison between two groups of patients after colostomy closure.

	Purse-string	Linear	p-value
Duration of hospitalization (days)	6.4±1.1	15.5±4.6	0.0001
POSAS (observer)	17.0±0.8	20.3±8.2	0.038
POSAS (parents)	17.1±5.0	23.6±7.8	0.045
Colostomy closure (days)	84.26±0.9	95.20±1.1	0.029
Wound healing	17.9±5.2	27.8±8.1	0.034
Scar length (mm)	24.09±0.1	52.15±1.0	0.033
Wound infection (%)	0	10	0.022

POSAS: Patient and Observer Scar Assessment Scale.

Early complication was seen in 2 (n=1) and 16% (n=8) of patients in purse-string and linear closure, respectively (p=0.014). In linear group, eight cases showed early complications, including wound infection (n=5) and anastomose dehiscence (n=3).

**Figure 1:**
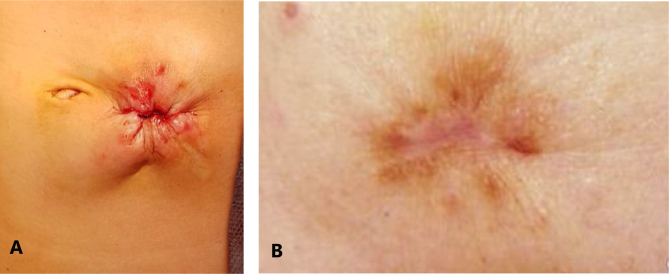
Surgical wound following colostomy closure using purse-string method at (A) 0 day and (B) 1 year.

**Figure 2:**
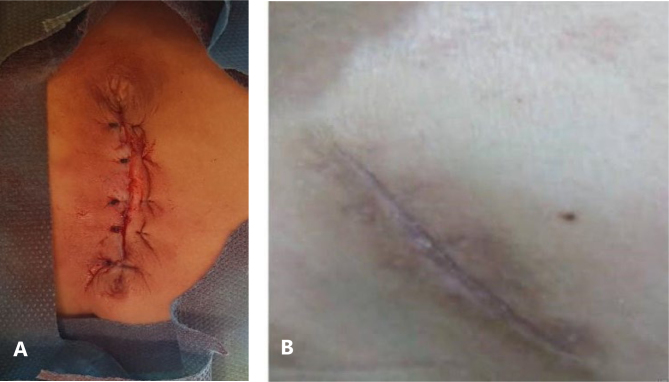
Closure using linear technique at (A) 0 day and (B) 1 year.

Scar length was 24.09±0.1 mm in purse-string and 52.15±1.0 mm in linear group (p=0.033, Figures 1 and 2). The POSAS scores for observer and parents are mentioned in Table 2.

## DISCUSSION

Several techniques have been used for ostomy closures since many years. Anorectal malformation was the most common cause of the colostomy in our study, which is consistent with the findings of Bischoff et al.^2^ which showed mortality in ostomy closure^2^.

Wound infection is one of the frequent complications^12,19^. In this study, site infection was not seen in purse-string method compared to 10% in linear technique, which is similar to the results of Dusch et al.^6^ which showed no infection in purse-string against 24% in linear technique. Purse-string method was associated with lower rate of wound infection compared to linear ostomy closures^9,14^. Also, Marquez et al. showed less wound infection in purse-string method compared to linear method^17^. In contrast, Milanchi et al.^19^ and Sutton et al.^24^ reported that wound infection was not seen following purse-string ostomy closure.

In addition, Vermulst et al.^26^ and Lee et al.^14^ found that there was no significant difference between purse-string and linear techniques in terms of surgical wound infection.

Cosmetic outcome was more favorable in purse-string compared to linear closure, which is similar to the findings of Hsieh et al.^10^ and Sutton et al.^24^.

Ostomy wound healing was significantly shorter in purse-string method compared to linear method, which is consistent with some studies^21^.

In this study, duration of purse-string technique was less than linear technique, which is similar to the study by Dusch et al.^6^.

Patient satisfaction was higher in the purse-string group compared to the linear group, which is similar to the study by Hajibandeh et al.^8^.

This study has some limitations. It is a single-center study. Sample size was the main limitation of this study.

## CONCLUSION

The purse-string closure of the colostomy is the safe method with favorable cosmetic appearance, less frequent wound infection, and less duration of colostomy closure. It is recommended to study with more sample size and more follow-up period in future.
